# Intensive care patients eligible for intermediate care unit-level care: a single-centre prospective cohort study in Belgium

**DOI:** 10.62675/2965-2774.20260185

**Published:** 2026-01-08

**Authors:** Jérôme Tack, Arnaud Bruyneel, Julie Maes, Gwennaëlle Mercier, Fabio Silvio Taccone, Magali Pirson

**Affiliations:** 1 Université Libre de Bruxelles School of Public Health Hospital Management and Nursing Research Department Brussels Belgium Health Economics, Hospital Management and Nursing Research Department, School of Public Health, Université Libre de Bruxelles - Brussels, Belgium.; 2 Clinical Research and Translational Unit Grand Hospital of Charleroi Charleroi Belgium Clinical Research and Translational Unit, Grand Hospital of Charleroi - Charleroi, Belgium.; 3 Helora Mons Hospital Constantinople and Warquignies Site Nursing Department Mons Belgium Nursing Department, Helora Mons Hospital Constantinople and Warquignies Site - Mons, Belgium.; 4 Université Libre de Bruxelles Hôpital Universitaire de Bruxelles Intensive Care Unit Brussels Belgium Intensive Care Unit, Hôpital Universitaire de Bruxelles, Université Libre de Bruxelles - Brussels, Belgium.

**Keywords:** Workload, Nursing, supervisory, Intensive care units, Hospitalization, Hospitals

## Abstract

**Objective:**

To estimate the number of intensive care unit hospitalization days that could have been managed at an intermediate care unit level and to assess the impact of intermediate care unit-eligible patients on intensive care unit-free days, hospital-free days, and nursing workload.

**Methods:**

This single-center, prospective cohort study included all patients admitted to the intensive care unit of an academic hospital between June 1, 2021, and May 31, 2022. All adult (> 18 years of age) patients with an intensive care unit stay exceeding 24 hours were eligible for inclusion. Data from a total of 1,547 patients were analysed. Daily, intensive care unit head nurses identified patients eligible for intermediate care unit management based on predefined criteria. Nursing workload was quantified using the Nursing Activities Score, and 16,478 Nursing Activities Score assessments, recorded at the end of each nursing shift, were collected.

**Results:**

A total of 1,457 intensive care unit hospitalization days (16.7% of the total) were classified as eligible for intermediate care unit-level management. An increase in cumulative intermediate care unit days was significantly associated with fewer intensive care unit - and hospital-free days (adjusted incidence rate ratio = 0.98 [95%CI 0.97 - 0.99] for both). A strong negative correlation was observed between the monthly proportion of intermediate care unit-eligible intensive care unit days and the intensive care unit occupancy rate (R = −0.703, p = 0.011). By shift, the median Nursing Activities Score for intensive care unit patients was 72.4 [59.6 - 87.5] compared to 63.5 [52.6 - 72.8] for intermediate care unit-eligible patients in the morning, 71.5 [58.1 - 86.6] *versus* 56 [47.8 - 66.1] in the afternoon and 66.1 [53.5 - 81.1] *versus* 53.9 [45 - 64.7] during night shifts (p < 0.001 for all).

**Conclusion:**

This study highlights the potential impact of early identification of intermediate care unit-eligible patients on optimizing the use of intensive care unit beds and improving the organization of patient care. A lower Nursing Activities Score might help select patients for intermediate care unit care, supporting the clinical relevance of such a score in the daily assessment of intensive care unit patients.

## INTRODUCTION

Strained capacity in the intensive care unit (ICU) is conceptually defined as a mismatch between ICU resource availability and the demand for high-quality care and admission for critically ill patients.^(1)^ This strain on ICU capacity has been associated with delayed admissions,^(2,3)^ increased rates of healthcare-associated infections,^(4)^ unplanned ICU readmissions^,(5)^ and prolonged ICU length of stay (LOS).^(6)^ Moreover, receiving treatment in an ICU during periods of high capacity strain has been associated with an increased risk of patient mortality; ICU strain is influenced by multiple patient-related and system-level factors, including the availability of ICU beds and the criteria used for ICU admission.^(1,2,7)^

In Belgium, despite having one of the highest ICU bed-to-population ratios in Europe (17.4 beds per 100,000 inhabitants),^(8,9)^ ICU occupancy rates were already high before the pandemic, ranging from 75 to 90%.^(10-12)^ The severe acute respiratory syndrome coronavirus 2 (SARS-CoV-2) pandemic significantly disrupted ICU organization and bed availability across Europe.^(13-16)^ In January 2022, at the peak of the health crisis, 192 ICU beds (9.6% of available beds) were unavailable in Belgium. This restricted capacity arises in the context of increasing demand for ICU beds, a trend expected to continue in the coming years.^(17-19)^ It is therefore crucial to explore strategies to alleviate ICU bed occupancy constraints and ensure sufficient capacity for optimal care of critically ill patients.

Intermediate care units (IMCs) offer a viable solution to this problem. Intermediate care units are designed for patients who do not require an ICU's intensive medical and nursing resources but still need more advanced care than that provided in general wards.^(20)^ In recent years, IMCs have been increasingly utilized as hospitals expand their intensive care services to manage complex patients, optimize resources, and reduce costs. For example, in Spain, the number of IMCs increased from 16 before the pandemic to 41 afterwards,^(13)^ while in Japan, IMCs grew by 32% between 2016 and 2022.^(21)^ Additionally, IMCs offer various advantages, including reducing daily healthcare costs and shortening hospital stays.^(22-24)^

Recent published studies have emphasized the fact that adequate nurse staffing is essential to guaranteeing quality patient care and preserving patient well-being.^(25)^ However, the literature remains inconsistent regarding the ideal nurse to patient ratio (N/P) in IMCs, with reported values ranging from 1:1 to 1:11.^(23,26,27)^ Belgium has no standardized national policy governing IMCs. The legally mandated N/P, set at 1:3 in ICUs^(28)^ and 1:30 in general wards,^(29)^ are frequently exceeded in practice.^(12,30)^

The development of IMCs in Belgium offers a strategy for meeting the growing challenges of caring for critically ill patients. However, assessing the potential workload associated with caring for patients eligible for care in the IMC is crucial. This study aimed to estimate the number of ICU hospitalization days that could have been managed at an IMC level and to assess the impact of IMC-eligible patients on ICU-free days, hospital-free days, and nursing workload.

## METHODS

### Study design

This single-center prospective cohort study was conducted, including all patients admitted to the five ICUs of a Belgian academic hospital in Brussels. The Department of Intensive Care is comprised of 30 beds (5 for each unit) without specialized areas that accommodate various ICU patients (e.g., cardiac surgery, neurosurgery, extracorporeal membrane oxygenation [ECMO]). The strengthening of reporting of observational studies in epidemiology (STROBE) checklist for cohort studies was applied to guide and verify the completeness and transparency of the reporting process.^(31)^

### Participants

Patients were recruited over 1 year, from June 1, 2021, to May 31, 2022. All patients admitted to the ICU for more than 24 hours were included. Pediatric patients (< 18 years) were excluded.

### Intermediate care unit criteria

Each day between 8 a.m. and 10 a.m., ICU head nurses identified patients eligible for the IMC. The criteria used to identify patients who were eligible for the IMC were developed using a Delphi method, a structured communication technique designed to achieve consensus among experts. This process was conducted with a panel of five experienced physicians and six ICU head nurses, selected for their expertise in critical care. The Delphi method was carried out in two iterative rounds, with a 1-month interval between each session. In the first round, participants independently reviewed and rated proposed criteria based on existing literature.^(32)^ Their responses were then analyzed, and a summary of the findings was shared with the panel. In the second round, participants were asked to reassess their ratings, considering the group's feedback, to refine and finalize the criteria. The criteria selected were: the patient is not intubated, does not require continuous veno-venous hemofiltration (CVVH), has no arterial catheter or cardiac output monitoring (e.g., PiCCO, Swan-Ganz), has no active bleeding (maximum of 2 units of red blood cells per day), and does not require sedation. Post-operative patients were excluded from the IMC for the first 48 hours. Patients with an external ventricular drain (EVD) could be admitted to the IMC if the other criteria were met.

### Variables and data encoding

In this study, two variables were significant concerning the study objectives. First, ICU-free days and hospital-free days were calculated as these are increasingly chosen as primary or secondary endpoints for critically ill patients.^(33-35)^ This indicator measures the number of days alive outside the ICU or hospital. Traditional "duration" endpoints (e.g., LOS) can be misleading, as an intervention that reduces mortality may paradoxically lengthen the average LOS or duration of mechanical ventilation.^(35)^ The calculation of 30-day ICU-free days and 90-day hospital-free days has been frequently used in ICU clinical trials.^(34,36)^

Intensive care unit data were used to calculate ICU-free days. ICU-free days were defined as 30 minus the number of days spent in the ICU (range: zero to 30 days). For patients who survived and remained in the ICU for less than 30 days, ICU-free days were determined by subtracting the ICU LOS from 30. Patients who died before or on day 30 were assigned an ICU-free days value of zero. Similarly, patients with an ICU LOS of 30 days or more were also assigned zero ICU-free days.

A similar approach was applied to calculate hospital-free days at 90 days. Hospital-free days were defined (range: zero to 90 days). For patients who survived and were discharged from the hospital before day 90, hospital-free days were determined by subtracting the total hospital LOS from 90. Patients who died before or on day 90 were assigned a hospital-free days value of zero. Patients with a hospital LOS of 90 days or more were also assigned zero hospital-free days. This method accounts for the prolonged recovery and potential complications as 90 minus the total hospital LOS associated with extended hospitalizations in critically ill patients.^(33,35,37)^ Practically speaking, the higher the ICU-free days and hospital-free days scores, the better the outcome for the patient.

Second, nursing workload was evaluated. Several tools have been developed to assess ICU workload. The Nursing Activities Score (NAS) is considered the most appropriate tool, providing an objective measure.^(38,39)^ The NAS and its French guidelines published in 2019 were used for this study.^(40,41)^ Nurses recorded the NAS at the end of each shift. In February 2021, nursing teams received a 1-hour training on NAS, including theory and practical exercises. A trial period from March 1, 2021, to May 31, 2021 ensured proper NAS recording. Guides and tutorials were available, and head nurses received additional training to support NAS coding and verify data accuracy. The NAS was recorded daily at each shift change, and 16,478 NAS records were collected. Data were collected via Epimed Monitor®, with some information extracted from electronic patient records.

In addition, data for several other variables included in the study were collected. At admission, the following variables were recorded: age (years), sex, type of admission (medical, emergency surgery, scheduled surgery), source of admission (emergency room, operating room, transfer from other hospital, ward/floor), and the Simplified Acute Physiology Score 3 (SAPS 3).^(42)^ This data was routinely encoded by the medical team. At discharge, LOS in ICU and in hospital (days) were collected, and patients were followed after hospital discharge for mortality. The occupancy rate was calculated each day based on the number of patients present in the ICU at 8 a.m. and the number of beds available.

### Statistical analysis

Gaussian continuous variables are presented as means ± standard deviation (SD). Non-Gaussian continuous variables are reported as medians with interquartile ranges (IQR). Unpaired Student's t-tests and Wilcoxon tests were used for mean and median comparisons. Pearson's χ² test assessed categorical data differences.

For the correlation analysis, Spearman's Rank-Order coefficient (rho) was used to assess the relationship between occupancy rate and the percentage of IMC patients by month. The interpretation of correlation strength in this study follows published references.^(43)^

Two distinct models were used to assess associations between ICU-free days, hospital-free days, and IMC-days. Crude and adjusted relative rate ratios (RRR) with 95% confidence intervals (95%CIs) were derived from multinomial regressions, while crude and adjusted incidence rate ratios (IRR) with 95%CIs were obtained from negative binomial regressions. The Wald Chi² test P-value was reported. Due to overdispersion, negative binomial regression was applied to model count data related to LOS. Multivariate models were adjusted for all variables included in the univariate analysis. Confounding variables were selected based on existing literature and available data. Model adequacy was assessed using the Hosmer-Lemeshow test, and the variance inflation factor was evaluated to avoid collinearity.

For the multivariable analysis, candidate variables were selected based on previously reported associations with ICU and hospital LOS in the literature and the availability of relevant data in the study database. A multivariable regression model was developed using a forward stepwise selection procedure, with a predefined entry criterion of a p value < 0.05 at the univariate analysis. Variables were entered sequentially according to their univariate association with the outcome, starting with the strongest. The selection process was terminated when no additional variable met the inclusion criterion. Although commonly used in clinical research, this approach should be interpreted cautiously, as stepwise procedures may lead to overfitting and biased estimates if applied in small samples or without external validation.^(44)^ Analyses were conducted using Software for Statistics and Data Science (18.0), with p values < 0.05 considered statistically significant.

### Ethical considerations

Each patient received a randomly assigned anonymous identifier. Ethical approval was obtained from the Erasme Hospital Ethics Committee on May 20, 2021 (P2021/262/B4062021000139). In accordance with the ethics committee's requirements, an information notice explaining the study and its objectives was displayed at the entrance of the department throughout the entire data collection period. As approved by the ethics committee, formal informed consent was waived.

## RESULTS

**Figure 1:**
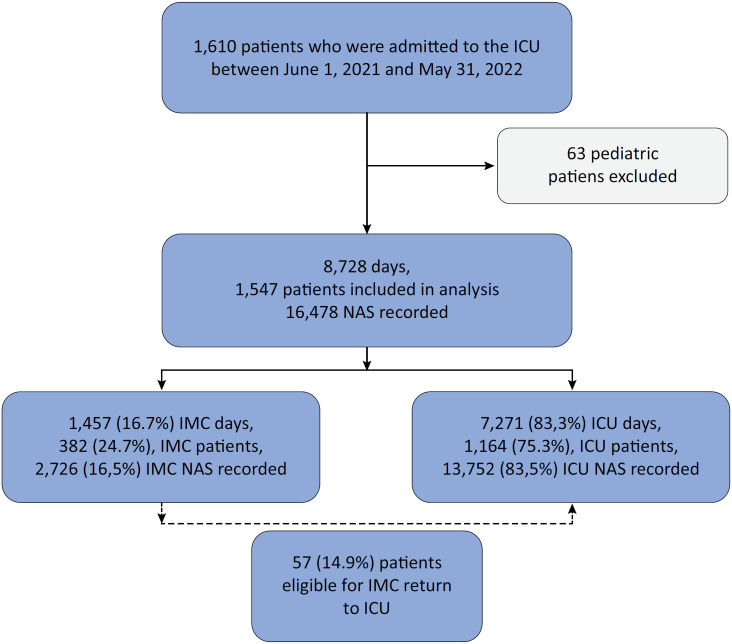
Patient flow chart.

Of the 1,547 patients included in this study (Figure 1), 382 (24.7%) were determined to have at least one IMC-eligible day during their stay, representing 16.7% of the hospitalization days included in the study. A total of 14.9% of patients initially deemed eligible for IMC care required return to ICU care (Figure 1). Of the 1,547 patients analyzed, 916 (59.2%) were male, with a mean age of 58.8 years (± 16.7). The median LOS in the ICU was 2 days. The characteristics of the study population are presented in table 1.

ICU - intensive care unit; NAS - Nursing Activities Score; IMC - intermediate care unit.

**Table 1:** Sociodemographic data

		Total (n = 1,546)	IMC patients (n = 382)	ICU patients (n = 1,164)	p value
Patient characteristics					
	Men	916 (59.2)	223 (58.4)	693 (59.5)	0.689
	Age (years)	58.8 ± 16.7	59.2 ± 17.1	58.7 ±15.9	0.559
	Length of stay ICU (days)	2 [4]	3 [3]	2 [4]	< 0.0001
	Length of stay hospital (days)	10 [20]	11 [21]	10 [19]	0.890
	ICU-free days	25 [6]	25 [5]	26 [6]	< 0.0001
	Hospital-free days]	10 [20]	11 [22]	10 19]	0.552
	ICU mortality	204 (13.2)	51 (13.4)	153 (13.1)	0.943
	Hospital mortality	284 (18.4)	68 (17.8)	215 (18.5)	0.736
	SAPS 3	42 [25]	41 [27]	42 [24]	0.765
Type of admission					
	Emergency surgery	158 (10.3)	42 (11.0)	116 (10.0)	< 0.0001
	Medical	905 (58.4)	213 (55.8)	692 (59.4)	
	Scheduled surgery	483 (31.3)	127 (33.2)	356 (30.6)	
Source of admission					
	Emergency room	278 (18.0)	71 (18.5)	207 (17.8)	0.078
	Operating room	607 (39.3)	152 (39.8)	455 (39.1)	
	Transfer from other hospital	296 (19,1)	69 (18.2)	227 (19.5)	
	Ward/Floor	365 (23,6)	90 (23.5)	275 (23.6)	

IMC - intermediate care unit; ICU - intensive care unit; SAPS - Simplified Acute Physiology Score. Results expressed as n (%), mean ± standard deviation or median [interquartile range].

### Intensive care unit occupancy and intensive care unit days

**Figure 2:**
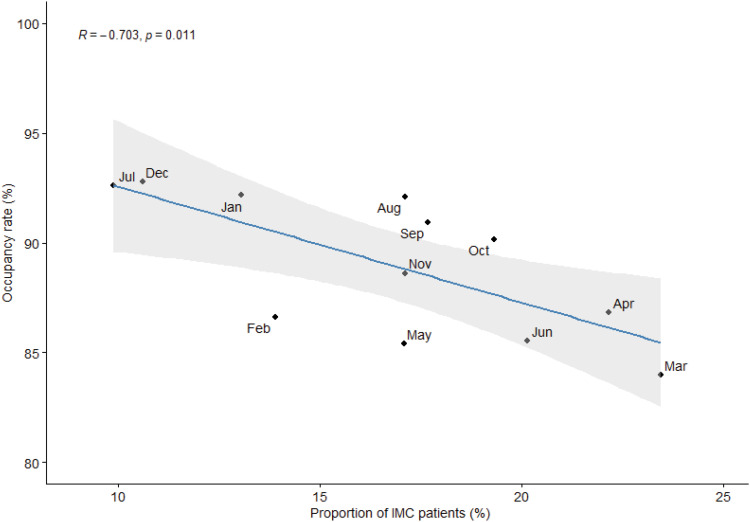
Correlation between proportion of intermediate care unit patients and occupancy rate, by month.

There was a strong negative correlation (R = −0.703, p = 0.011) between the proportion of ICU days detected each month and the ICU occupancy rate. The proportion of IMC-eligible days varied from month to month. A direct relationship was observed between ICU occupancy rates and the number of days that could be transferred to an IMC. Specifically, December, January, and July had the highest occupancy rates and the lowest number of IMC transferable days. Conversely, March had the lowest occupancy rate and the highest number of ICU days that could be transferred to an IMC (Figure 2).

IMC - intermediate care unit.

### Workload assessment

**Figure 3:**
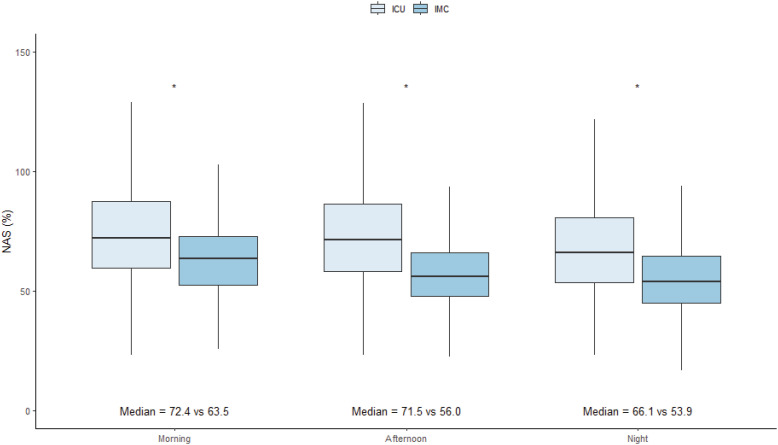
Comparison of intensive care unit and intermediate care unit patients workloads according to Nursing Activities Score, by shift.

Across all shifts, ICU patients consistently had significantly higher NAS scores compared to IMC-eligible patients. For the morning shift, the median NAS for ICU patients was 72.4 [59.6 - 87.5], whereas the median NAS was 63.5 [52.6 - 72.8] for IMC-eligible patients (p < 0.001). For the afternoon shift, ICU patients had a median NAS of 71.5 [58.1 - 86.6], compared to 56.0 [47.8 - 66.1] for IMC-eligible patients (p < 0.001). For the night shift, the median NAS for ICU patients was 66.1 [53.5 - 81.1], while IMC-eligible patients had a median NAS of 53.9 [45 - 64.7] (p < 0.001; Figure 3).

ICU - intensive care unit; IMC - intermediate care unit; NAS - Nursing Activities Score. * p value < 0.001.

### Intensive care unit - and hospital-free days

Analysis of associations between factors revealed that an increase in cumulative IMC-eligible days was significantly associated with a higher risk of fewer ICU- and hospital-free days (adjusted IRR = 0.98 [95%CI 0.97-0.99] for both). Older patients were at a slightly higher risk of having fewer ICU-free and hospital-free days (adjusted IRR = 1.01 [95%CI 1.00 - 1.02]). Higher SAPS 3 scores were significantly associated with a higher risk of fewer ICU and hospital-free days (adjusted IRR = 0.98 [95%CI 0.97 - 0.99] for both). Emergency surgery patients were at higher risk of having fewer ICU- and hospital-free days (adjusted IRR = 0.91 [95%CI 0.88 - 0.93]), while medical admissions also demonstrated a higher risk, with fewer ICU and hospital-free days (adjusted IRR = 0.92 [95%CI 0.90 - 0.94] and 0.87 [95%CI 0.86 - 0.89], respectively). Finally, women were at higher risk of having fewer ICU- and hospital-free days compared to men (adjusted IRR = 0.96 [95%CI 0.94 - 0.98] and 0.95 [95%CI 0.93 - 0.96], respectively) (Table 2).

**Table 2:** Analysis of variables independently associated with ICU- and hospital-free days, according to patient characteristics

Factors		ICU-free days				Hospital-free days			
		IRR crude (95%CI)	p value	IRR adjusted (95%CI)	p value	IRR crude (95%CI)	p value	IRR adjusted (95%CI)	p-value
IMC cumulative days		0.98 (0.97 - 0.99)	0.004	0.98 (0.97 - 0.99)	0.032	0.98 (0.97 - 0.99)	< 0.001	0.98 (0.97 - 0.99)	< 0.001
Age (year)		0.99(0.98 - 1.01)	0.099	1.01 (1.00 - 1.02)	< 0.001	0.99 (0.98 - 0.99)	< 0.001	1.01 (1.00 - 1.02)	< 0.001
SAP 3 score		0.98 (0.97 - 0.99)	< 0.001	0.98 (0.97 - 0.99)	< 0.001	0.98 (0.97 - 0.99)	< 0.001	0.98 (0.97 - 0.99)	< 0.001
Type of admission									
	Scheduled surgery	REF		REF		REF		REF	
	Emergency surgery	0.93 (0.89 - 0.96)	< 0.001	1.02 (0.95 - 1.06)	0.342	0.81 (0.79 - 0.83)	< 0.001	0.91 (0.88 - 0.93)	< 0.001
	Medical	0.77 (0.75 - 0.79)	< 0.001	0.92 (0.90 - 0.94)	< 0.001	0.68 (097 - 0.69)	< 0.001	0.87 (0.86 - 0.89)	< 0.001
Gender									
	Male	REF		REF		REF		REF	
	Female	0.99 (0.97-1.01)	0.534	0.96 (0.94 - 0.98)	< 0.001	0.99 (0.98 - 1.01)	0.965	0.95 (0.93 - 0.96)	< 0.001

ICU - intensive care unit; IRR - incident rate ratio; IMC - intermediate care; SAPS - Simplified Acute Physiology Score.

## DISCUSSION

This study identified ICU patients who could have been managed in an IMC and evaluated their impact on ICU-free days and the nursing workload associated with these patients. Our analysis revealed that 16.7% of ICU patient-days were potentially eligible for IMC-level care. Furthermore, the analysis of ICU-free and hospital-free days suggests that the accumulation of IMC-eligible days may contribute to more extended overall hospital stays. These patients were also associated with a significantly lower nursing workload, with NAS scores consistently higher for ICU patients across all shifts compared to IMC-eligible patients. Several key findings emerged from this study, consistent with recent literature and have important implications for the organization and management of critical care resources.

During the study period, 16.7% of ICU patient-days were identified as eligible for care in the IMC, with a strong correlation between monthly ICU occupancy rates and the proportion of IMC-eligible patients. This suggests that patients who no longer require ICU care are more likely to remain in the ICU during periods of lower ICU occupancy. This phenomenon may also reflect Belgium's lack of standardized ICU admission and discharge criteria. This observation is in line with the findings from other studies that reported that, in the absence of strict criteria for ICU admission, a significant number of ICU patients could be treated at a lower level of care.^(11,45)^ This could also be a consequence of a fee-for-service financing model fragmented by department. Additionally, staffing norms in Belgian ICUs are based on the number of available beds (N/P of 1:3 at maximum) rather than the actual number or severity of patients. As a result, an empty ICU bed may be perceived as a financial loss, potentially contributing to the high ICU occupancy rates observed in several Belgian studies.^(12)^ These findings highlight opportunities for optimizing resource utilization. The establishment of IMCs has been shown to provide numerous benefits for patient care, including reduced hospital bed occupancy and lower staffing costs.^(18,22)^ The financial argument for keeping patients in the ICU is counterbalanced by the impact on ICU-free days and hospital-free days. Indeed, the longer IMC-eligible patients remain in the intensive care unit, the worse their outcomes are in terms of ICU-free and hospital-free days. Keeping these patients in the ICU to maintain an adequate occupancy rate increases the risk of a reduced number of survival days without hospitalization.

To our knowledge, this is the first study to highlight this relationship. This phenomenon could be explained by delayed specialist management and the well-documented risks associated with prolonged ICU LOS. These include extended exposure to invasive devices (central and arterial catheters, urinary catheters),^(46)^ ICU-acquired weakness leading to greater dependency after discharge,^(47,48)^ and factors such as delirium, cognitive impairment,^(49)^ and post-intensive care syndrome, partly due to continuous exposure to a stressful medical environment.^(49)^ However, it is important to emphasize that our study evaluated eligibility for IMC care rather than the actual effectiveness of IMC implementation. The limited global adoption of IMCs may be partly attributed to uncertainty regarding their actual clinical and organizational benefits and the complexities introduced by varying healthcare financing models. Furthermore, a publication bias may favor reports of successful IMC initiatives, which are often context-dependent and not universally generalizable. Consequently, our findings should be interpreted within the specific framework of assessing patient eligibility rather than directly endorsing IMC implementation.

In addition, any redesign of healthcare delivery systems, including introducing IMCs, may result in unintended consequences. For example, the availability of IMCs could potentially reduce hospital discharge rates or delay end-of-life care discussions, ultimately prolonging hospital LOS. Although our analysis focuses solely on patient eligibility, adding another transitional step in the continuum of care may lead to extended hospitalizations. These considerations underscore the need for rigorous impact evaluations when implementing IMCs, and future research should aim to identify strategies to mitigate such potential risks.

This study identified 16.7% of patients as suitable for IMC care. This percentage is lower than that reported in a previously conducted retrospective multicenter study in Belgium over a short period, where the number of IMC-eligible days detected based on nursing activity was higher.^(8)^ This difference may be explained by the fact that the hospital in this study is a tertiary academic hospital with highly specialized staff and equipment, providing regional services and regularly receiving referrals from primary and secondary hospitals. The 16.7% identified as suitable for IMC could be underestimated. Indeed, the selection criteria for this study excluded surgical patients as eligible for IMC during the first 48 postoperative hours. However, the literature reports the management of such patients in IMCs.^(26)^ This exclusion is justified by our estimation that the expertise of caregivers in the ICU is essential to rapidly detect risks of clinical deterioration.

The NAS was systematically higher for ICU patients, across all shifts, compared to those identified as IMC eligible. This finding indicates that patients eligible for IMC-level care require less intensive nursing care, further supporting their transfer to a lower-intensity environment. French researchers have recently assessed the workload of inpatients in their IMC.^(30)^ At 47.4%, their median NAS was lower than this study's. This difference could be explained by the fact that, in our study, patients eligible for an IMC are in the ICU, which may impact the workload of caring for these IMC patients (e.g., systematic rounds every 2 hours may not be necessary). Our results suggest an N/P ratio of 1:2 to 1:3, but this ratio must be refined when an IMC is opened. Nevertheless, this ratio is in line with several international recommendations.^(22,30-32)^

The results of this study also show that the establishment of an IMC requires careful consideration of the workload on ICU nursing staff. The creation of these units would result in the transfer of lower-workload patients out of the ICU, concentrating the most critical and high-dependency patients in the ICU.^(50)^ This reorganization could increase pressure on ICU staff, especially since current N/P ratios in ICUs are already considered outdated and insufficient to ensure quality care.^(12)^ Such a concentration of high-dependency patients risks increasing the likelihood of staff burnout, compromising care quality, and jeopardizing patient safety.^(51)^ Thus, implementing an IMC cannot be considered without a comprehensive review of the organization of critical care in Belgium. This reorganization should include a reassessment of staffing standards, adaptation of hospital infrastructure, and ongoing training of healthcare personnel to meet the needs of critically ill patients.^(52,53)^ The experience of other countries that have integrated IMCs into their healthcare systems could also serve as a solid foundation to guide this reform.^(27,54)^

Among patients initially considered suitable for IMC-level care, 14.9% required transfer back to ICU-level care. This finding underscores the importance of continuous monitoring and the need to further refine critical care eligibility criteria to ensure patient safety and optimal resource utilization. Nursing workload should also be taken into account, as high workload at the time of ICU discharge has been associated with an increased risk of ICU readmission.^(55)^ Incorporating well-defined clinical parameters, particularly those related to cardiovascular, neurological, renal, and metabolic status, within a graded care model could help ensure that patients are directed to the most appropriate level of care for their clinical condition.^(26)^ Establishing precise and dynamic criteria for transferring patients between care units is essential for balancing workloads while maintaining the quality and safety of care.^(26,27)^

This study highlights the potential benefits of IMCs for reducing pressure on ICUs by transferring eligible patients to the IMC. Moving patients eligible for IMC care out of the ICU should positively impact outcomes in terms of ICU-free days and hospital-free days, optimizing patient care and improving healthcare efficiency. By accurately identifying patients likely to be transferred to the IMC, hospitals can increase ICU capacity for the most critically ill patients, improve overall patient flow, and potentially reduce healthcare costs by shortening hospital LOS. However, the observed rate of patients returning to the ICU after an IMC transfer suggests that patient selection criteria and monitoring protocols need to be refined. Implementing standardized guidelines and using advanced monitoring technologies could improve decision-making processes for patient transfers.

### Limitations and future research

While this study provides valuable insights, several limitations must be acknowledged. First, its single-center design and the specific criteria used to determine IMC eligibility limit the generalizability of the findings. Future multicenter studies, involving diverse patient populations and a variety of IMC organizational models, are necessary to validate and extend these results. Additional analyses should also explore the influence of institutional factors such as hospital size, patient demographic and pathological profiles, N/P, and ICU bed availability, which may affect both IMC eligibility assessment and clinical outcomes.

One of the key strengths of this study lies in its prospective design and the daily collection of NAS scores, which enabled a robust and dynamic evaluation of nursing workload. However, a potential limitation is the risk of immortal time bias, which may arise if specific periods - during which outcomes such as ICU discharge or death could not occur - are incorrectly attributed to a given exposure group, potentially leading to overestimating associations between exposure and outcomes. Moreover, our database did not include daily assessments of illness severity, limiting the ability to identify progressive clinical deterioration that may have contributed to prolonged ICU stays and increased IMC-eligible days. As such, the observed IMC-eligible days may reflect the overall trajectory of illness rather than serve as an independent predictor of adverse outcomes.

Although the study highlights the potential economic advantages of reallocating patients to IMC beds to optimize ICU resource utilization, no formal cost-effectiveness analysis was performed, limiting the ability to quantify the actual financial benefits. Future research should incorporate targeted economic evaluations to assess the cost savings associated with IMC implementation. Furthermore, the development of predictive models that integrate patient acuity, comorbidities, and real-time clinical data could refine IMC eligibility criteria and reduce the rate of unplanned transfers back to the ICU.

## CONCLUSION

This observational study suggests that intermediate care units have the potential to enhance the efficient use of intensive care unit resources and improve the organization of patient care. Our analysis identified a significant association between the cumulative number of intensive care unit days eligible for intermediate care unit-level care and a reduction in intensive care unit - and hospital-free days; however, this finding should be interpreted with caution due to the limitations inherent in the adjusted model. Patients eligible for intermediate care unit care were consistently associated with a lighter nursing workload, supporting the relevance of the selection criteria applied in this study. Future efforts should focus on evaluating and refining patient triage protocols and integrating advanced monitoring systems and predictive tools to optimize the role of intermediate care units within critical care pathways.

## Data Availability

The data is anonymised and kept securely by the authors.
